# Autohydrogenotrophic Denitrification Using the Membrane Biofilm Reactor for Removing Nitrate from High Sulfate Concentration of Water

**DOI:** 10.1155/2018/9719580

**Published:** 2018-08-05

**Authors:** Yanhao Zhang, Haohan Zhang, Zhibin Zhang, Yuchen Wang, Taha Marhaba, Jixiang Li, Cuizhen Sun, Wen Zhang

**Affiliations:** ^1^School of Municipal and Environmental Engineering, Shandong Jianzhu University, Jinan 250101, China; ^2^Co-Innovation Center of Green Building, Jinan 250101, China; ^3^John A. Reif, Jr. Department of Civil & Environmental Engineering, New Jersey Institute of Technology, Newark, NJ 07102, USA; ^4^Sustainable Technology Research Center, Shanghai Advanced Research Institute, Chinese Academy of Sciences, Shanghai 201210, China

## Abstract

This study investigated the performance of an autohydrogenotrophic membrane biofilm reactor (MBfR) to remove nitrate from water with high sulfate concentrations. The results of simulated running showed that TN removal could be over than 98.8% with the maximum denitrification rate of 134.6 g N/m^3^ d under the conditions of the influent sulfate concentrations of 300 mg SO_4_^2−^/l. The distribution ratio of H_2_ electron donor for nitrate and sulfate was 70.0 : 26.9 at the high influent loading ratio of sulfate/nitrate of 853.3 g SO_4_^2−^/m^3^ d : 140.5 g N/m^3^ d, which indicated that denitrification bacteria (DB) were normally dominated to complete H_2_ electron with sulfate bacteria (SRB). The results of molecular microbiology analysis showed that the dominated DB were *Rhodocyclus* and *Hydrogenophaga*, and the dominated SRB was *Desulfohalobium*, under the high influent sulfate concentrations.

## 1. Introduction

Nitrate-contaminated river or groundwater occurred everywhere in the world because the fertilizers were utilized extensively and part of the wastewater from industries was discharged randomly, especially in developing countries [1, 2]. The high concentrations of nitrate in drinking water (>10 mg N/l) would have a high risk to produce nitrosamines and cause methemoglobinemia, which was harmful to people's health [3, 4]. Therefore, a lot of methods to reduce nitrate from water sources have been reported [5, 6].

The effective methods to reduce nitrate include ion exchange [7] and reverse osmosis [8–10]. Due to the high cost of physiochemical technologies, their applications are limited in some extent [11]. The two normal types of the biological treatment are heterotrophic denitrification and autotrophic denitrification [12, 13]. The cost of the heterotrophic denitrification is high because the organic materials need often to add the carbon source for bacteria in the process which are low in groundwater [14, 15]. There are lots of advantages of autohydrogenotrophic technology, such as clear with hydrogen, low cost, and without secondary pollution [16, 17].

Recently, a new technology of hydrogen- (H_2_-) based membrane biofilm reactor (MBfR) has developed and got a good effect, which used autohydrotrophic bacteria in the denitrification processes [16, 18, 19]. The oxidized pollutants, such as SO_4_^2−^, CrO_4_^2−^, AsO_3_^−^, TCE, ClO_4_^−^, BrO_3_^−^, and SeO_4_^2−^, could be reduced by MBfR using H_2_ as electron donors [20–23]. While NO_3_^−^ and SO_4_^2−^ are chemical oxyanions that normally coexist in a variety of waters. There are many reasons caused NO_3_^−^ and SO_4_^2−^ coexisting in water, such as anthropogenic activities related to overusing of fertilizers and wastewater discharges, natural mineralogy related to SO_4_^2−^ minerals, and atmospheric deposition of NO_3_^−^ and SO_4_^2−^ [24]. On the other hand, in MBfR, the autohydrogenotropic bacteria could utilize NO_3_^−^ and SO_4_^2−^ as electron acceptors to generate energy for their growth [25], and several sulfate-reducing bacteria (SRB) are able to use alternative terminal electron acceptors to reduce sulfate such as nitrate [26].

The following equations could describe the stoichiometry of hydrogenotrophic denitrification and sulfur-reducing:

(1) $$\begin{array}{c} 2{{NO}_{3}}^{-}+2H^{+}+5H_{2}\to N_{2}+6H_{2}O\\ 4H_{2}+{{SO}_{4}}^{2-}\to H_{2}S+2H_{2}O+2OH^{-} \end{array}$$

While in some sites in the world (e.g., natural mineralogy), the contents of sulfate could be as high as hundreds or thousands micrograms per liter in the groundwater, which is used as a drinking water. Because SO_4_^2−^ is not normally considered a health concern, and no MCL has been established for SO_4_^2−^, so many references of autohydrogenotrophic denitrification could concern about sulfate reduction, but the concentrations of SO_4_^2−^ were relatively lower in the influents for research [27].

The aim of this study was to investigate the performance of autohydrogenotrophic denitrification under the high concentrations of sulfate by a hollow fiber membrane bioreactor with polyvinyl chloride (PVC) membrane.

## 2. Materials and Methods

### 2.1. Reactor in the Study

**Figure 1:**
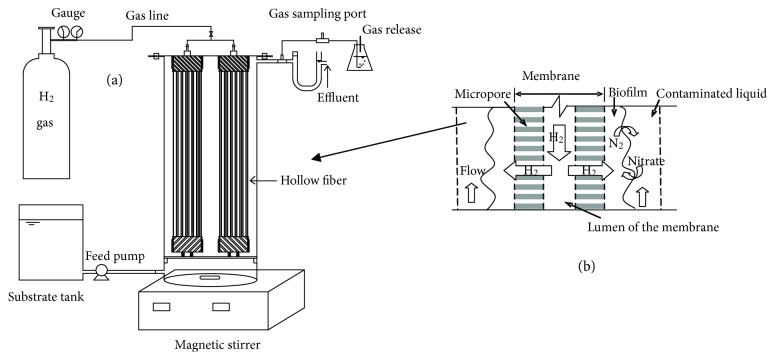
MBfR in the experiment (a) and theoretical views of MBfR (b).

The theory of denitrification using hydrogenotrophic bacteria is shown in Figure 1(b); the denitrification attached on the outside surface of membrane would utilize the H_2_ transferred from the lumen of the membrane at some extent of pressure to accomplish the denitrification. For the reactor, we use a transparent plastic cylinder to hold two membrane modules, and the influent fluid was flowed from upper side to the lower outlet, and the flow rate was controlled by a peristaltic pump (longer BT50-1J, Baoding, PRC), and the membrane made of polyvinyl chloride membrane with hydrophobicity alloy fiber was used in the study. The detailed schematic of the reactor could be seen in Figure 1(a). Also, the parameters of the membrane and the reactor are listed in Table 1.

### 2.2. Influent Water Source and Experimental Conditions

In the study, the influent water was taken from the sulfate- and nitrate-contaminated groundwater in the vegetable land at the suburb of Qingzhou (Weifang, China), where a lot of fertilizer had been used in the lands. The shallow groundwater around the vegetable land had been contaminated by nitrate and sulfate, and the water quality is shown in Table 2.

We stated up the reactor by inoculating the biofilm microorganisms from other MBfRs running for hydrogenotrophic denitrification for years in our lab. For simulating the different concentrations of sulfate in the influent water, some dosage of FeSO_4_·7H_2_O was fed in the influent pumped from the actual groundwater. The detailed experimental design of the reactor running could be seen in Table 3.

All the fluid samples collected in the experiments were kept at 4°C until the samples were analyzed. The NO_3_^−^-N, NO_2_^−^-N, and SO_4_^2−^ were measured by the ion chromatography (Dionex ICS 3000). The H_2_ unutilized by the denitrifiers would go into the headspace of the reactor. A GC 14-B equipped with a TCD detector (Shimadzu Co.) was used to test the H_2_ gas concentration in the headspace in the reactor by pumping gas from the gas port by a syringe, and the hydrogen content in the liquid could be calculated by Henry's law.

### 2.3. Sampling for Biofilm and the Analysis of Microbiology

In the experiments, at different running periods for the reactor, the biofilm would be sampled to analyze the changes of the microbial communities. For our study, when the water quality in the effluent was steady, that is, at day 40, day 80, and day 150, the biofilm samples were collected. According to our previous research, DNA extractions, PCR, and DGGE were done; see the detailed methods in [28]. As for the nucleotide sequencing, the reamplified DNA products were analyzed by Sangon Company (Shanghai, China). Shannon-Wiener index was used to analyze the diversity changes of microbial communities in different running periods of the reactor. The relation and the dendrogram generation among the biofilm bacteria in different running periods were calculated and analyzed by cluster analysis through the NTSYS-pc (2.10, Exeter Software, USA).

## 3. Results and Discussion

### 3.1. Operation and Effluent Quality of MBfR

**Figure 2:**
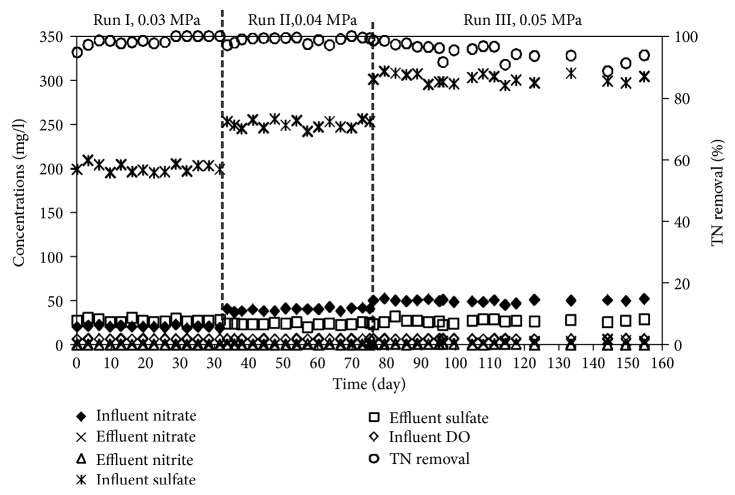
The water quality in the influent and effluent and TN removal.

In the beginning of the experiment, the biofilm established on the out surface of the fiber was only taken 3 days just because of the inoculation of bacteria from the reactors running over than years. Then, the reactor was operated over 155 days to evaluate the performance of MBfR under different conditions. The performance of MBfR over the operation periods was illustrated in Figure 2.

As shown in Figure 2, the influent concentrations of nitrate and sulfate ranged from 10–50 mg N/l and 100–300 mg SO_4_^2−^/l through the experiments, respectively. In the whole experiment period, the averages of TN removal were 96.4 ± 2.3%, 98.8 ± 1.0%, and 94.9 ± 2.8% in the Run I, Run II, and Run III, respectively. As for the water quality in the effluent, the averages of nitrate concentrations in the effluents were 0.7, 0.3, and 2.1 NO_3_^−^-N mg/l, for Run I, Run II, and Run III, respectively. And for nitrite in the effluent, the contents of nitrite in Run I are not detected, but were 0.2 and 0.4 NO_2_^−^-N mg/l, in Run II and Run III, respectively. It suggested that the high concentrations of sulfate have some extent inhabitation to denitrification in MBfR processes.

### 3.2. Performance of MBfR under High Concentration of Sulfate

In this experiment, the high sulfate concentrations up to 300 mg/l in the influent were used to investigate the performance of MBfR. Under the conditions of the different contents of sulfate in the influent, the denitrification loadings and sulfate loadings could be seen in Table 4.

The volumetric denitrification rates were changed from 55.7 g N/m^3^ to 134.6 g N/m^3^ with a good TN removal over than 94.9%, which was mainly caused by increasing the influent nitrate loadings. The sulfate reduction rate was changed from 155.4 to 266.7 g SO_4_^2−^/m^3^, which was not mainly controlled by the influent sulfate loading of 566.3–853.3 g SO_4_^2−^/m^3^, and the average sulfate removals were about 23.5–27.4%. It indicated that the nitrate would be utilized preferentially by denitrification bacteria (DB) than sulfate utilized by SRB in completion with H_2_ in MBfR, and nitrate respiration is energetically more favorable than sulfate respiration [31].

In the autohydrogenotrophic denitrification in MBfR, the SRB also utilized hydrogen as electron donor to reduce sulfate to sulfide; therefore, there would be a competition for hydrogen between the reductions of nitrate, sulfate, and other electron acceptors. The distributions of hydrogen electron in electron acceptors at different influent sulfate contents in this study and references are shown in Table 5. The calculations of the hydrogen electron's distributions in MBfR were according to our previous research [30]. Distributions of hydrogen electron were not only dependent on the concentrations of electron acceptors but also on the types of electron acceptors. But the distribution ratio of H_2_ on sulfate would be high as its concentration increases at the same conditions. As for sulfate in this study, even the influent sulfate loading increased gradually, the sulfate removal was contained at steady figure of about 25%, while the TN removal was almost over 95%, which can be seen from the distribution of electron-equivalent fluxes that the ratio of nitrate : sulfate was 70.0% : 26.9% (Run III). It indicated that DB could get more H_2_ than SRB whatever of the acceptor influent loading changes. While Table 5 also indicated that the high influent sulfate concentrations or high ratio of influent sulfate concentration to influent nitrate concentration would lead SRB to get more power in the competition for hydrogen among the electron acceptors, which could be used to select the special bacteria in MBfR operations for minimizing sulfate reduction [32].

### 3.3. H_2_ Utility

The effluent H_2_ concentrations in Runs I–III were very low, from 0.10 to 0.52 mg/l, which indicated that the H_2_ could be transferred well without bubble from the PVC membrane and be used sufficiently by DB and SRB; meanwhile, the system got an effective removal of nitrate.

The % unutilized hydrogen was calculated according to (2), that is, the part of H_2_ leaving out of reactor: the part utilized by bacteria. The H_2_ utility in the reactor is shown in Table 6.

(2) $$\begin{array}{c} \%H_{2}unutilized=100\%\times \frac{S_{H,o}}{0.143\left(S_{3,i}-S_{3,o}\right)+0.214\left(S_{3,i}-S_{3,o}-S_{2,o}\right)+0.083\left(S_{4,i}-S_{4,o}\right)+0.125\left(S_{5,i}-S_{5,o}\right)+S_{H,o}}, \end{array}$$

where the detailed meanings of *S*_3,i_, *S*_3,o_, *S*_2,o_, *S*_5,i_, *S*_5,o_, and *S*_H,o_ could be seen in [30].

As shown in Table 6, the sum of hydrogen utilization efficiency over the 3 periods was 97.7–99.5%; the remains may go into the effluent or out of the water. Among the sum of the H_2_ utility, nitrate got much more quota than that of sulfate and oxygen.

### 3.4. Analyses of Microbial Community

**Figure 3:**
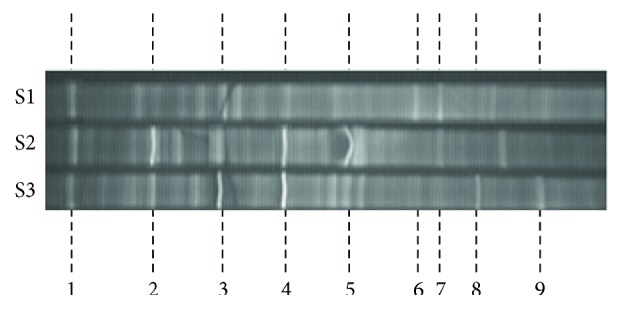
DGGE and on the day 40 (S1), day 80 (S2), and day 150 (S3) (the Arabic numerals meant the different dominated bands in the operation of MBfR).

The microbial communities in each running period of the reactor could be seen in the analyses of the DGGE (Figure 3). The DGGE indicted the dominant bands. Even the operation period was long in each running stage with different concentrations of sulfate in the influent, while the autohydrogenotrophic bacteria growth was very slow and the change of microbial community was considerately slow. In the beginning period of Run I, the bands were not clear and complicated, which indicated that the biofilm needs acclimation furthermore. While several bands, which were clear and simple, could be seen in Run II and Run III. The special bands with number 2, 3, and 4 in DGGE which were dominated were cut and sent to be sequenced. The results indicated that the bacteria in bands 2, 3, and 4 were similar to *Rhodocyclus*, *Hydrogenophaga*, and *Desulfohalobium*, with the similarity of 99%, 98%, and 99%, respectively. The *Rhodocyclus* and *Hydrogenophaga* were normal autotrophic bacteria, belonging to beta divisions within the Proteobacteria. This is consistent with our previous study [28]. The *Desulfohalobium* was found in Runs II and III, which is a Gram negative, anaerobic, sulfate-reducing, moderately halophilic, and rod-shaped bacterial genus from the family of Desulfovibrionaceae. This indicated that the SRB could be abundant with the influent concentration increasing and could enhance its strength of competition with nitrate for H_2_ [31].

## 4. Conclusion

The study investigated the performance of MBfR to remove nitrate companied with high influent concentrations of sulfate over 155 days. The results indicated that even in high concentration of sulfate in influent, the MBfR also could get a good denitrification effect with nitrate and nitrite under the US standard. The analysis of the molecular microbiology showed that microbial community structures of Runs II and III were similar, simple, and stable. The bacteria species of Betaproteobacteria which include *Rhodocyclales* and *Hydrogenophaga* were dominant DB for nitrate removal. The *Desulfohalobium* was found to be a dominant SRB in Runs II and III under the high concentrations of sulfate. The results would give some directions on the actual application of MBfR to remove nitrate or other oxidations in the drinking water.

## Figures and Tables

**Table 1:** The parameters of the reactor.

Parameters	Unit	Value
Numbers of fiber module		2
Outer diameter of fiber	cm	0.15
Inner diameter of fiber	cm	0.085
Fiber number in the reactor		96
Length of fiber	mm	140
Volume of fibers	cm^3^	23.74
Available surface area	cm^2^	633.34
Available volume of reactor	cm^3^	560
Void ratio	%	95.76
Specific surface area	m^2^/m^3^	113.10
Height	cm	22.0
Section area of reactor	cm^2^	28.26
Diameter of reactor	cm	6.0
Available volume of reactor	cm^3^	560

**Table 2:** Water quality parameters of the groundwater.

Total dissolved solids (mg/l)	pH	Alkalinity (mg/l as CaCO_3_)	Hardness (mg/l as CaCO_3_)	DO	Nitrate (mg N/l)	Nitrite (mg N/l)	Sulfate (mg/l)
300–400	7.2~7.5	320~500	400~650	6.0–6.4	35~60	ND	250~450

ND: not detected.

**Table 3:** Experimental design of the reactor running.

	Start-up	Run I	Run II	Run III
Running time (day)	3	1–40	41–80	81–155
H_2_ pressure in the fiber (MPa)	0.02	0.03	0.04	0.05
Nitrate concentration in the influent (mg N/l)	10.0 ± 2.0	20.0 ± 2.0	40.0 ± 4.0	50.0 ± 4.0
Sulfate concentration in the influent (mg/l)	100 ± 10.0	200 ± 10.0	250 ± 10.0	300 ± 10.0
Flow rate (ml/min)			1.1	
HRT (h)			8.5	

**Table 4:** The influent loadings and volume reductions for nitrate and sulfate under different influent sulfate concentrations.

Influent sulfate contents (mg/l)	Influent sulfate loading (g/m^3^ d)	Volume sulfate reduction (g/m^3^ d)	Nitrate loading (g N/m^3^ d)	Volume denitrification rate (g N/m^3^ d)	Sulfate in effluent (mg/l)	Nitrate in effluent (mg N/l)	References
200	566.3	155.4	57.8	55.7	145.3	0.7	This study
250	707.3	166.3	112.5	111.6	191.3	0.3	This study
300	853.3	226.7	140.5	134.6	221.5	2.1	This study
42	118.5	50.7	56.5	55.5	24	0.3	[29]
92	262.6	109.6	139.5	133.8	54	2	[29]
78	216.8	85.3	141.7	136	46.5	2	[30]

**Table 5:** Distributions of hydrogen electron in electron acceptors at different influent sulfate contents.

Influent sulfate (mg/l)	Influent nitrate (mg N/l)	Nitrate (%)	Sulfate (%)	Oxygen (%)	Cr (VI) (%)	References
200	20	57.9	36.1	6.0		This study
250	40	71.8	24.4	3.8		This study
300	50	70.0	26.9	3.1		This study
42	20	76.0	15.9	8.1		[29]
92	50	81.2	15.2	3.6		[29]
78	50	87.5	12.5			[30]
78	10	69.9	29.2		0.9	[33]
78	5	55.7	42.8		1.5	[33]

**Table 6:** The H_2_ utility in the MBfR.

	Sum of H_2_ utility (%)	H_2_ utility for nitrate (%)	H_2_ utility for sulfate (%)	H_2_ utility for O_2_ (%)
Run I	97.7	61.1	36.6	9.3
Run II	99.4	75.2	24.2	6.0
Run III	99.5	73.0	26.6	4.9

## Data Availability

The data used to support the findings of this study are available from the corresponding author upon request.
